# Tissue-specific transcriptomics reveals a central role of *CcNST1* in regulating the fruit lignification pattern in *Camellia chekiangoleosa*, a woody oil-crop

**DOI:** 10.48130/FR-2022-0010

**Published:** 2022-08-03

**Authors:** Chao Yan, Ziyan Nie, Zhikang Hu, Hu Huang, Xianjin Ma, Sijia Li, Jiyuan Li, Xiaohua Yao, Hengfu Yin

**Affiliations:** 1 State Key Laboratory of Tree Genetics and Breeding, Research Institute of Subtropical Forestry, Chinese Academy of Forestry, Hangzhou, Zhejiang 311400, China; 2 Key Laboratory of Forest Genetics and Breeding, Research Institute of Subtropical Forestry, Chinese Academy of Forestry, Hangzhou, Zhejiang 311400, China

**Keywords:** *Camellia chekiangoleosa*, Fruit lignification, NAC transcription factor, Lignin biosynthesis, Transcriptomics

## Abstract

Fruit lignification is of significant economic importance because it affects the quality of fruit and the production of seed oil. The specified lignification pattern in* Camellia* *chekiangoleosa* fruits plays critical roles in its seed oil yield, but little is known about how this lignification process is regulated. Here, we report on a comprehensive tissue-specific transcriptomics analysis conducted for *C.* *chekiangoleosa* fruit. By mining the differentially expressed genes, we found that lignin biosynthesis and transcriptional regulation pathways were significantly enriched in the lignified tissues. The homolog of NST-like transcription factor, *CcNST1*, was highly expressed in lignified seed coat and endocarp tissues; transgenic analyses of *CcNST1* in *Arabidopsis* and hybrid poplar revealed the enhanced lignification levels of various tissues. Gene expression analysis of the transgenic lines uncovered potential downstream genes involved in the regulation of lignin biosynthesis. This work provides a valuable gene expression resource and identified the pivotal role of *CcNST1* in regulating the lignin biosynthesis underlying fruit lignification.

## INTRODUCTION

Lignification is a crucial process of fruit development that plays various roles in different types of fruits^[1]^. In angiosperms, the lignification of the pericarp is essential for seed protection and their dispersal. In the dry-fruit crop walnut, the extensive lignification of endocarp (known as the nutshell) is a key economic trait; impaired development of the nutshell often adversely affects the quality of walnut kernel, leading to malformed seed kernels. In fleshy fruits, the lignin process is mostly overlooked or considered negligible; however, unexpected lignification of fruit tissues could be caused by disease and stress conditions, which can affect the taste and quality of fruits^[2]^.

The accumulation of lignin leads to the lignification of fruit, which is essential for maintaining the integrity of the plant cell wall and resisting plant pathogens^[3]^. During the hardening process of the pericarp, lignin conjugates with the cellulose and hemicellulose network to provide rigidity and tensile strength to secondary walls, in a process similar to wood formation^[4]^. Lignin is a type of secondary metabolite derived from phenylpropane. After a series of biochemical reactions, including deamination, hydroxylation and methylation, phenylalanine is finally transformed into three types of lignin monomers^[2]^. These are then separately polymerized to form p-hydroxyphenyl lignin, guaiacyl lignin, and syringyl lignin^[5]^. The lignin biosynthesis pathway in plants has been extensively investigated. The enzymes involved in its two major processes — monolignol biosynthesis and monolignol polymerization — have been characterized through genetic and biochemical studies^[6]^. A cascade of conserved core enzymes is fundamental for the biosynthesis of lignin in diverse plant species^[7,8]^.

In recent years, several transcription factors (TFs) have been identified which control the plant lignification process via direct or indirect regulation of lignin biosynthesis genes. The transcription regulation network of lignin biosynthesis, which is primarily based upon studies of *Arabidopsis thaliana* and *Populus trichocarpa*, is highly complex, with extensive feedback among different types of transcription factors^[9,10]^. Various studies have shown that plant-specific NAM, ATAF and CUC (NAC) and Myeloblastosis (MYB) TFs play important roles in the regulation of secondary cell wall biosynthesis in *A. thaliana* and* P. trichocarpa*^[11]^. In *A. thaliana*, the genes *MYB58* and *MYB63* encoded transcriptional activators of the lignin biosynthetic pathway^[12]^. In *P. trichocarpa*, those of *PtrMYB28*, *PtrMYB4*, *PtrMYB3* and *PtrMYB20* found to activate lignin biosynthesis during the wood formation process^[13,14]^. The *NAC SECONDARY WALL THICKENING PROMOTING FACTOR* (*NST*) genes were demonstrated to function as master regulators for initiating the lignin biosynthesis during secondary cell wall formation^[11]^. In *A. thaliana*, the loss-of-function mutants of* nst1 nst3/snd1*, and *nst1 nst2 nst3/snd1* impaired lignin biosynthesis in xylem and interfascicular fiber cells^[15,16]^. Further, *NST1*, *NST2*, and *NST3* were found to directly target the expression of downstream TFs including *MYB46*, *SND3*, *MYB103*, and *KNAT7*^[9,17]^.

The formation pattern of fruit lignification requires the timely coordination of multiple types of TFs^[18]^. In *A. thaliana*, specific fruit lignification is indispensable for seed dispersal^[19]^. Three MADS-box TFs, namely *FRUITFUL* (*FUL*), *SHATTERPROOF1* (*SHP1*) and *SHATTERPROOF2* (*SHP2*), were shown to determine the formation of fruit dehiscence zone^[20]^. Later, the FUL-SHP regulatory module was found conserved across different plant species to control the expression of key genes involved in fruit development ^[21−24]^. In fruit crops, several key TFs involved in lignin biosynthesis have been identified and characterized. *EjMYB8*, a MYB family TF from loquat, activates the expression of lignin biosynthesis genes, including *EjPAL1*, *Ej4CL1*, and *Ej4CL5*, through direct binding to their promoters^[25]^. In citrus, overexpression of *CsMYB85* significantly increases the expression of *Cs4CL1*, leading to a greater lignin content of fruits^[26]^. In pear, *PpNAC187*, a NST homolog, operates as an important regulator of stone cell formation that directly activates the expression of lignin biosynthesis genes^[26]^. In loquat, *EjNAC1*, the NAC type TF, are able to activate the lignin biosynthesis genes in response to temperature changes during the postharvest storage period^[27]^.

Oil-Camellia is an important woody edible crop predominately cultivated in China. Camellia-oil refers to a suite of species in the genus *Camellia*, such as *C. oleifera*, *C. meiocarpa*, and *C. chekiangoleosa*, whose main purpose for cultivation is to produce seed oil. Currently, *C. oleifera* is the main one cultivated for Camellia-oil production^[28, 29]^. Nevertheless, *C. chekiangoleosa — *closely-related to *C. oleifera — *is emerging as a favorable cultivation plant because of its high-quality oil; further, the oil content of its seed kernel is 5%−10% higher than that of *C. oleifera*^[30]^. Unlike *C. oleifera*, *C. chekiangoleosa* fruits have a very low level of lignin, which plays a prominent role in regulating the rate of fruit expansion, the size of seeds, and seed oil production^[31]^. In the present study, a tissue-specific transcriptome analysis of *C. chekiangoleosa* was conducted to elucidate its fruit lignification pattern. Through gene expression analysis and functional verification of transgenic *A. thaliana* and poplar, the NAC domain transcription factor, *CcNST1*, was revealed as a key regulator of fruit lignin biosynthesis. This work presents a genome-wide gene expression profile underlying the patterning of fruit lignification and characterizes the functions of *CcNST1* in the regulation of fruit lignin biosynthesis.

## MATERIALS AND METHODS

### Plant materials

The experiment materials of* C. chekiangoleosa* were obtained from the Research Institute of Subtropical Forestry of the Chinese Academy of Forestry (RISF, CAF; Hangzhou City, Zhejiang Province, China; 119°57′22'' N, 30°03′30'' E). The flower buds and different tissues of the fruits (exocarp, mesocarp, endocarp, seed coat, and seed kernel samples) were collected and placed into liquid nitrogen and stored at –80 °C before use. To distinguish the stages of fruit growth, the materials of *C. chekiangoleosa* were collected from the Jinhua ‘Dongfanghong’ Forest Farm (Jinhua City, Zhejiang Province, China; 119°30′12'' E, 29°1′55'' N). *Arabidopsis thaliana* (Columbia ecotype) was grown and maintained in a growth chamber under an 8-h light/16-h dark photoperiod at 21 °C and 40% humidity. The hybrid poplar ‘Nanlin895’ (*Populus deltoides* × *P. euramericana* cv. ‘Nanlin895’) was obtained from the Nanjing Forestry University (Nanjing, Jiangsu Province, China) and preserved as cuttings in the greenhouse of RISF. The transgenic poplar plants were initially grown in the growth chamber for about 2 months and then transferred into the greenhouse.

### RNA preparation and sequencing

Total RNA of bud and each tissue was extracted using an RNAprep Pure Plant Kit (Tiangen, Beijing, China). The concentration and integrity of the total RNA were checked by a Nanodrop 2000 spectrophotometer (Thermo Fisher, CA, USA). The sequencing libraries were constructed using the TruSeq RNA library Prep Kit v2, after which transcriptome sequencing was carried out by an Illumina HiSeq4000 using the 2 × 150 bp sequencing pipeline. Both the library construction and sequencing were performed externally, by the Hangzhou LC-Bio Co., Ltd (Hangzhou, China). The raw reads were filtered to remove any low quality reads and adapter sequences, leaving only clean reads used for the assembly of unigenes of *C. chekiangoleosa* by Trinity v2.4.0^[32]^. All original sequencing reads were deposited into the National Center for Biotechnology Information (NCBI) SRA database, under Bioproject PRJNA565081. The transcriptome assembly of *C. chekiangoleosa* is available in the NCBI TSA database under accession number GISO00000000.

### Gene expression analysis

To identify the differentially expressed genes (DEGs), the expression levels of transcripts were calculated as reads per kilobase per million (RPKM). DESeq2 was used to identify DEGs according to two criteria: an absolute fold-change > 2 and FDR adjusted p-value < 0.05^[33]^. For real-time quantitative PCR (qRT-PCR) analysis, the total RNA was reverse-transcribed by the Prime Script RT reagent Kit (Takara, Dalian, China). The qRT-PCR was run on an ABI PRISM 7300 Real-Time PCR System (Foster City, CA, USA) which used SYBR Premix Ex Taq (Code No. RR420A, Takara, China); relative expression levels calculated according to the 2^−ΔΔCᴛ^ method^[34]^. The gene-specific primers were designed in PrimerExpress 2.0 software (Supplemental Table S1), and three biological replicates, each with 2 or 3 technical replicates, were used to quantify gene expression.

### Gene cloning and vector construction

Total RNA was reverse-transcribed by using a cDNA synthesis kit (Fermentas, Canada). To identify the homologs of NST-like genes, sequence alignments were performed using the NST1 (AT2g46770) protein sequence (BLASTp, e-value cutoff: E-15). Candidate transcripts were evaluated according to their sequences and expression profiles (Supplemental Table S2). Each full-length sequence was cloned by PCR amplification and then ligated to the T-vector pMD^TM^20 (Code No. 3270, Takara, Dalian, China) for its sequence verification. Then a CcNST1-Green Fluorescence Protein (GFP) fusion construct was obtained by cloning into the pEXT06/g vector (Cat. exv09, BIOGLE, Hangzhou, China), using specific corresponding primers (Supplemental Table S1). To construct the expression vector for poplar, the pEXT06/g-CchNST1-GFP plasmid was digested by *Bam*HI and *Pst*I and ligated into the pCambia2301 backbone.

### Transgenic analysis

The *Agrobacterium* *tumefaciens* strain C58 (pGV3101) harboring the constructs were transformed into *A. thaliana* by the floral-dip method^[35]^. Seeds of the ensuing T0 generation were collected and sown on 1/2 MS medium that contained 50 mg/L hygromycin. The independent T1 lines were verified by DNA amplification and gene expression analysis. The subcellular localization analysis was conducted using the root tips. The GFP signals were observed under a Zeiss LSM 800 confocal microscope. To observe the nucleus, each root sample was stain with 0.1 μg/mL DAPI (Sigma, Shanghai, China). The transgenic of hybrid poplar was performed according to the method as described by Kumar & Fladung ^[36]^.

### Histological analysis

Cross-sections of *Arabidopsis* and poplar tissues were prepared and stained with safranin and fast green, as previously described by Yin et al.^[37]^. The root and stem tissues were collected from the wild type and transgenic lines at ca. 38 d post-germination. Stem tissues were sampled between 0.5 and 1.0 cm in length to the basal area, and the mature zone of roots collected ca. 1 cm away from the root tip. For the analysis of poplar, its stem tissues were prepared using the middle part of the fourth internode. The lignin accumulation in fruits for different periods was observed by staining the cross section of fruits with phloroglucinol-hydrochloric acid^[15]^.

## RESULTS

### Tissue-specific transcriptomics revealed genes associated with fruit lignification in *C. chekiangoleosa*

**Figure 1:**
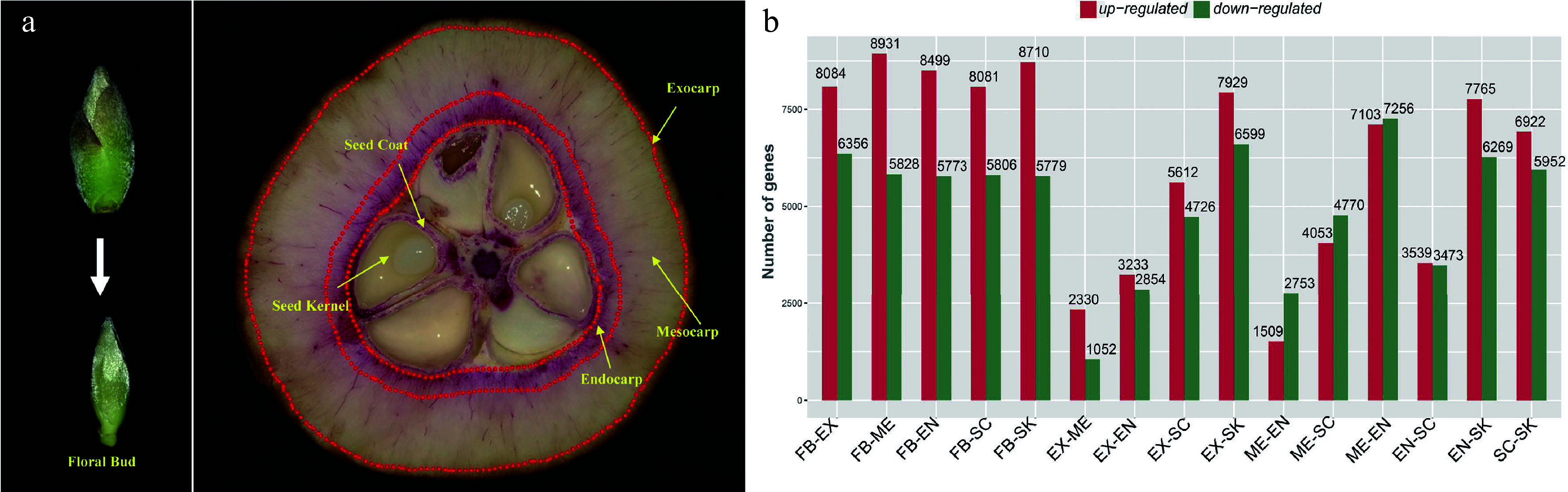
Tissue-specific transcriptomics in fruit of *Camellia chekiangoleosa*. (a) Morphology of tissues used for RNA sequencing. On the left is the incipient floral bud; its outside scale leaves are removed before a sample’s preparation. On the right are fruit tissue types at the stage of fruit enlargement; for each, three biological replicates were used for independent library construction and sequencing. FB, floral bud; EX, exocarp; ME, mesocarp; EN, endocarp; SC, seed coat; SK, seed kernel. The red-stained areas indicate the lignified tissues stained by phloroglucinol-hydrochloric acid. Yellow arrows indicate the tissues that were collected for sampling. Three biological replicates were used for library preparation and sequencing analysis. (b) Numbers of differentially expressed transcripts between tissue types. Red and green colors indicate the up-regulated and down-regulated genes, respectively, in each comparison.

In order to identify the genes involved in the lignification of fruits, we performed a detailed tissue-specific transcriptome analysis of *C. chekiangoleosa*. Six tissue types with three biological replicates were collected: floral bud (FB), exocarp (EX), mesocarp (ME), endocarp (EN), seed coat (SC), and seed kernel (SK), to determine the global expression patterning of genes (Fig. 1a). In the developmental stage of fruit expansion, both EN and SC were lignified (Fig. 1a) whereas EX and SK were not; a high level of lignified cells were present in SC and EN (Fig. 1a).

We obtained an average of ca. 79 million reads per RNA sequencing library for the *de novo* construction of the transcriptome (Supplemental Table S3). The assembled transcriptome consisted of 40,042 unigenes with a N50 value of 1,676 bp (Supplemental Table S4). The transcriptome assembly was annotated using multiple public databases; only those transcripts (unigenes) annotated in at least one database were retained for gene identification (Supplemental Fig. S1a–c). Next, the transcriptome was used as a reference to identify the DEGs). We first obtained the expression levels based on the mapping of RNA sequencing reads and then designated the transcripts with > 2-fold change in expression (False Discovery Rate [FDR] corrected p-value < 0.05) as DEGs. Many DEGs were detected between each comparison of different tissue types; in particular, EX-ME and EX-EN displayed relatively fewer DEGs (Fig. 1b), which was consistent with their tissue homology.

### Augmented lignin biosynthesis and its transcriptional regulation were essential for fruit lignification

**Figure 2:**
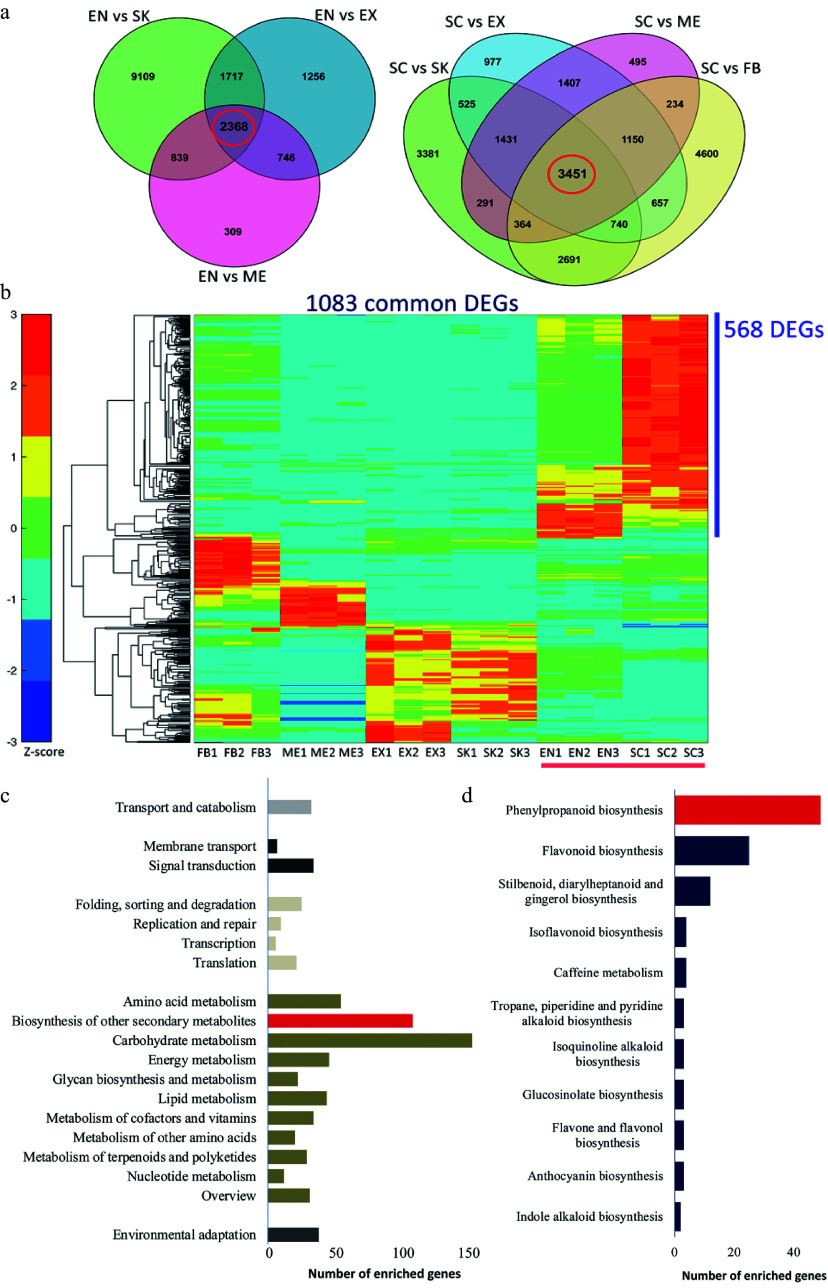
Functional characterization of differentially expressed genes (DEGs) that are involved in the lignification of the endocarp and seed coat of *Camellia chekiangoleosa*. (a) Venn diagrams of the DEGs in comparison to EN (left) and SC (right), which revealed 2348 and 3451 unigenes in the EN-group (left red circle) and SC-group (right red circle), respectively. (b) The EN-group and SC-group analysis yielded 1083 DEGs for gene expression analysis. The heatmap analysis of these 1083 genes identified clusters of them highly expressed in various tissue types. The red bar indicates the highly lignified EN and SC tissues. The blue bar indicates those genes highly expressed in EN and SC (568 DEGs); C, KEGG enrichment analysis of 568 DEGs that were highly expressed in EN and SC. D, Distribution of the number of genes that are enriched in 'Biosynthesis of other secondary metabolites'.

To analyze the expression pattern of genes associated with lignin accumulation in *C. chekiangoleosa*, EN and SC tissues were selected (due to their high lignin levels); these were also used to distinguish the pertinent genes in the lignification process (Fig. 2a). There were 2,368 and 3,451 common DEGs in the EN-group and SC-group, respectively (Fig. 2a). Integrative analysis revealed 1,083 common DEGs by comparing these two groups (Fig. 2b); in further evaluating the expression patterns of these common DEGs, 568 of them were highly expressed in SC and EN (Fig. 2b). The functional annotation of these highly expressed DEGs revealed that the KEGG pathway 'phenylpropanoid pathway' was significantly enriched, suggesting an early initiation of the lignin biosynthesis (Fig. 2c & d). This result yielded a pool of potential genes likely involved in the lignification of fruits.

**Figure 3:**
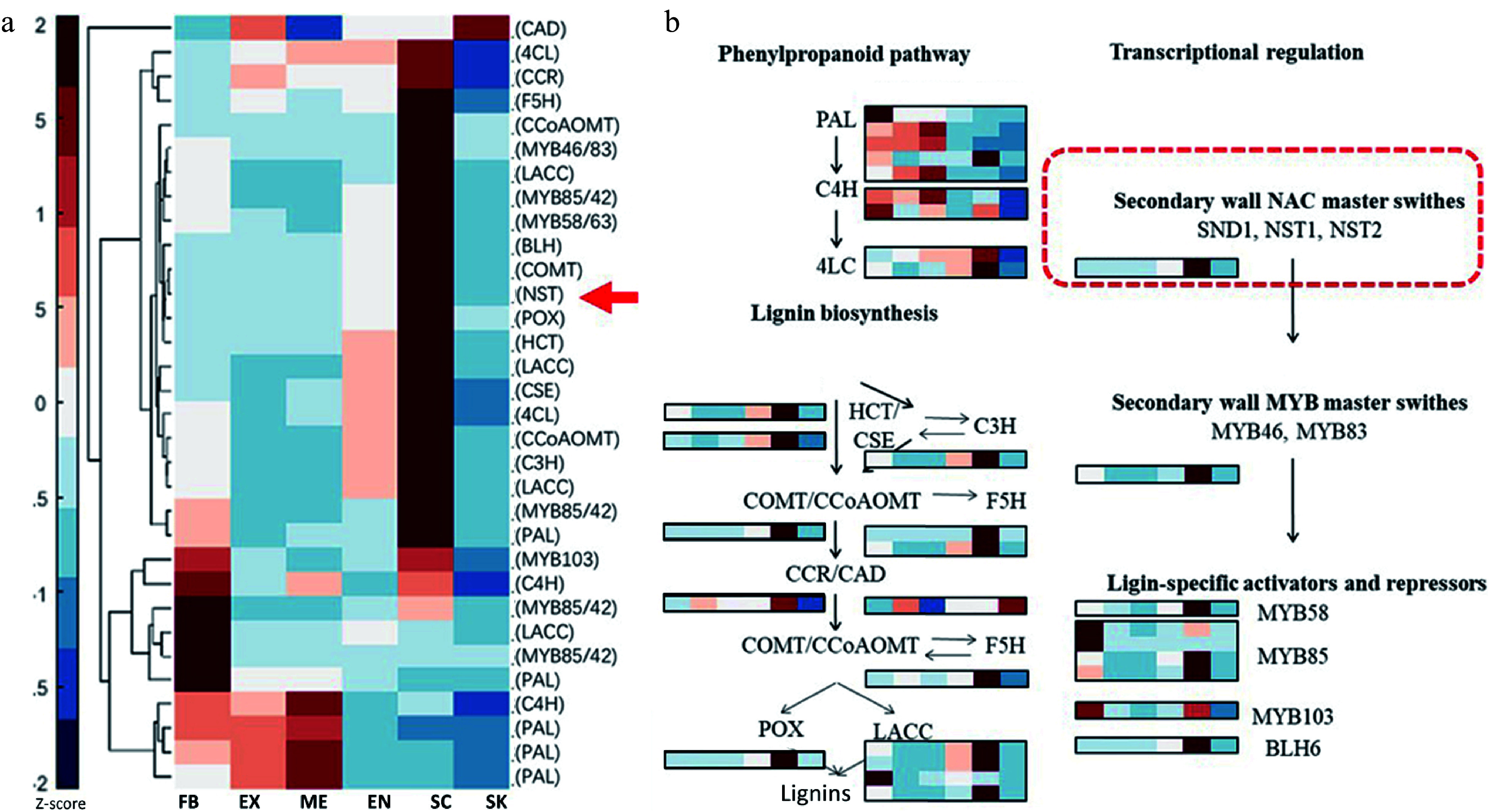
Tissue-specific expression analysis of lignin biosynthesis and transcriptional regulation gene in *Camellia chekiangoleosa*. (a) Heatmap of gene expression patterns for lignin-related genes that were identified based on sequence similarity. The gene symbols from *Arabidopsis* are used to indicate the potential homologs in *C. chekiangoleosa*. The red arrow indicates the NST homolog. Mean expression levels of transcripts are used for the expression analysis. (b) The key genes participating in lignin biosynthesis and its transcriptional regulation are presented according to known pathways identified in *Arabidopsis*. The master switch of secondary cell wall formation as regulated by NAC family TFs are highlighted in the red-dashed square.

Both the lignin biosynthesis pathway and its transcriptional regulation have been extensively studied^[15,18]^. Here, potential key genes involved in the regulation of lignin biosynthesis were identified based upon a sequence similarity analysis between *C. chekiangoleosa* and *A. thaliana* (Supplemental Table S5). Combined with the DEGs analysis, we screened out 15 lignin biosynthesis genes and six TF genes that could be involved in fruit lignification (Fig. 3a). The proposed biosynthesis and transcriptional regulation pathways were reconstructed to elucidate the lignification process in *C. chekiangoleosa* fruits (Fig. 3b). This revealed that different types of relevant transcription factors were possibly operating under a hierarchical regulatory network to induce lignin biosynthesis, among which the NAC transcription factor (NST ortholog) might direct a master switch given its intensive expression levels in both SC and EN tissue (Fig. 3b).

### Identification and expression profiling of *CcNST1* revealed a high positive correlation between the tissue lignin level and gene expression abundance

**Figure 4:**
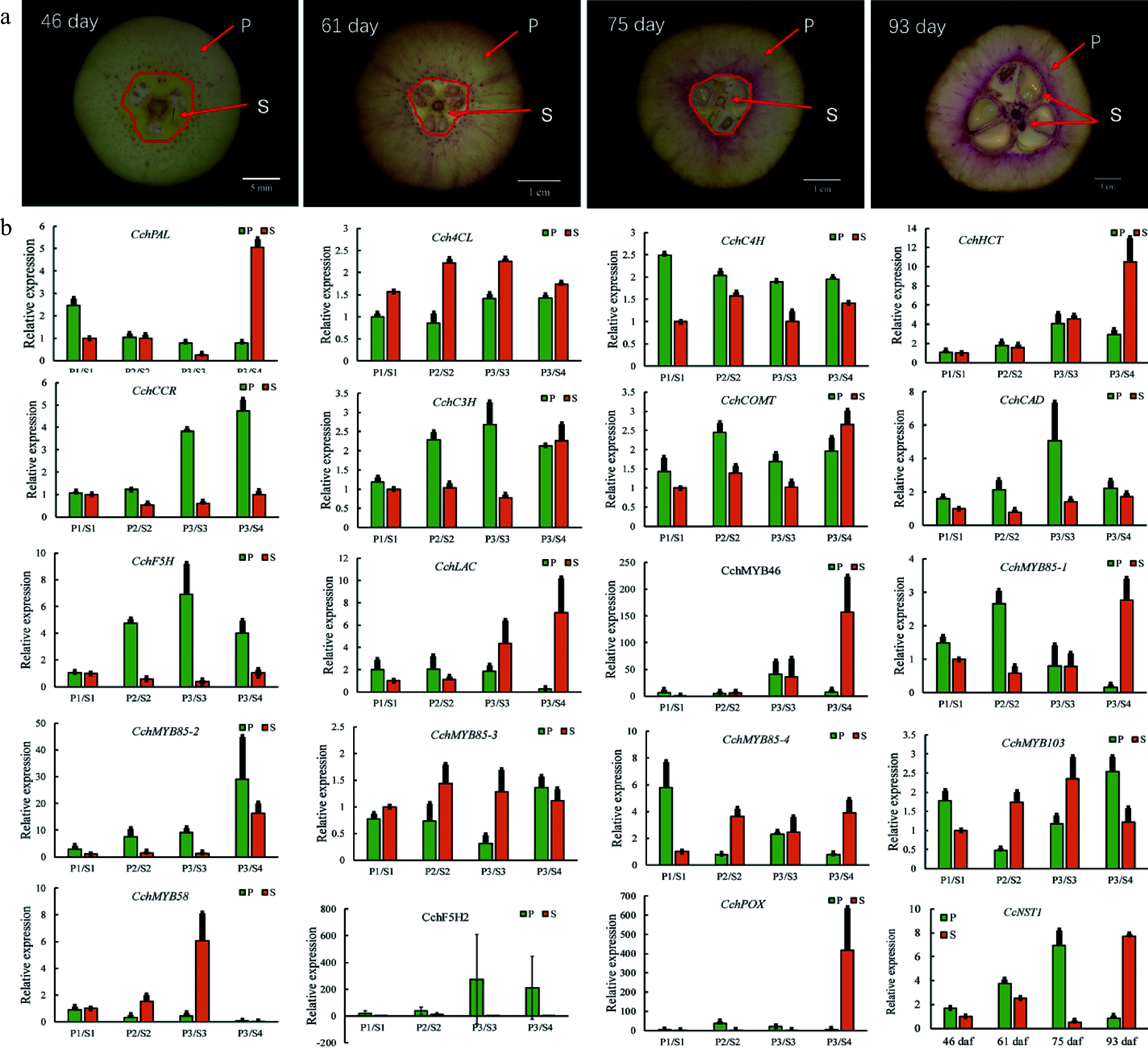
Expression analysis of genes involved in the regulation of lignin biosynthesis in pericarp and seed tissues during the fruit development of *Camellia chekiangoleosa.* (a) Staining of the vertical section of *C.* *chekiangoleosa* fruit in different periods. The purple stain signals from phloroglucinol-HCl indicate the lignified cells; the red arrowheads indicate the respective pericarp and seed tissue portions sampled at different stages of fruit development. The selection of the sampling is based on the developmental curves of *C. chekiangoleosa* fruits. P denotes mixed pericarp tissues; S denotes the mixed seed tissues. The arrows point to the areas of mixed tissues sampled at different developmental stages. (b) The qRT-PCR analysis of expression patterns of lignin biosynthesis genes at the four critical stages of P and S tissues. The expression of *CchNST1* was significantly up-regulated at 75 to 93 d post-fertilization in the P and S samples, corresponding to the lignification of the endocarp and seed coat. Values are means ± s.d. of three biological replicates.

To identify the key factors governing fruit lignification, we performed a gene expression analysis during the development of* C. chekiangoleosa* fruits by focusing on the establishment of lignified tissues (Fig. 4a). A growth curve of fruit development was derived based on the fruit size and lignification patterns (Supplemental Fig. S2). According to the levels of lignin, four critical stages of *C. chekiangoleosa* fruit development were discernible: *stage 1*, not lignified; *stage 2*, initiation of lignification; *stage 3*, fruit expansion and maintenance of lignification; *stage 4*, lignification completed (Supplemental Fig. S2; Fig. 4a). Next, the fruit pericarp (P) and seed-associated (S) tissues were dissected to verify the expression profiles of 20 candidate genes, including lignin biosynthesis and transcriptional regulators. The expression of these candidates agreed well with the transcriptomic results (Fig. 4b); notably, the *CcNST1* displayed high correlations with the degree of lignification of both the endocarp and seed coat (Fig. 4b). To further verify the contribution from the NST-like gene in *C. chekiangoleosa*, we evaluated the transcriptome and identified 15 potential NST homologs with full-length ORF (open read frame) (Supplemental Table S2; Supplemental Fig. S3b). We cloned the full-length coding regions of *CcNST1* and performed a phylogenetic analysis, which indicated that *CcNST1* was an ortholog of the *SND1/NST* gene (Supplemental Fig. S3a & c). These results suggested that *CcNST1* was an important regulator controlling the fruit lignification process in *C. chekiangoleosa*.

### Ectopic expression of* CcNST1* in *Arabidopsis* enhanced the lignification of tissues

**Figure 5:**
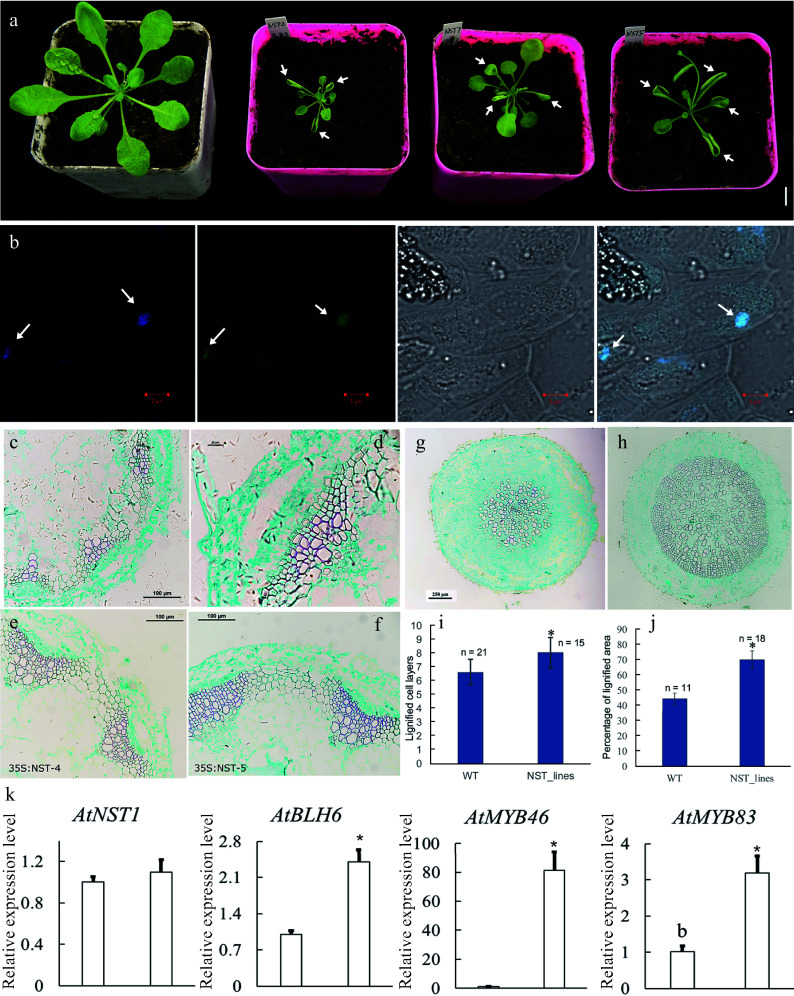
Overexpression of *CcNST1* in *Arabidopsis*. (a) Overexpression lines displayed various growth defects. Scale bar = 1 cm. The arrows indicate the striking curling leaves. (b) Subcellular localization analysis of the CcNST1:GFP fusion protein by confocal microscopy. Arrows indicate the signals in the nucleus. From left to right, the panels depict the DAPI signal, GFP signal, bright field, and superimposed images; scale bars = 5 µm. (c) & (d) Histological analysis of stem morphology in wild-type stems. (e) & (f) Histological analysis of stem morphology in the transgenic lines; scale bars = 100 µm. Histological analysis of root morphology in the transgenic lines (g) and (h) wild type; scale bars = 100 µm. (i) Statistical analysis of lignified cells in stem and root tissues. n indicated the independent measurements; values are means ± s.d.. (j) Statistical analysis of lignified areas in root tissues. The number of samples used for each statistical analysis is indicated by n. (k) Relative expression levels of *Arabidopsis NST1*, *BLH6*, *MYB46* and *MYB83* genes between the wild type and transgenic lines. Three independent transgenic lines were used for gene expression analysis. The expression of endogenous *AtNST1* was not significantly changed. Asterisks indicate significant p-values (< 0.05) for the Student’s t-test.

To investigate the roles of *CcNST1*, overexpression lines in *Arabidopsis* were generated. The T2 lines were identified via PCR using construct-specific primers (Supplemental Table S1; Supplemental Fig. S4a). Three lines displaying strong phenotypic alterations and high expression levels were used for further analyses (Supplemental Fig. S4b). We found that the overexpression lines displayed pleiotropic growth defects in different tissues, including a smaller size, upward curling of leaves, and distorted stems (Fig. 5a). Histological analysis of the wild type versus transgenic lines was carried out to understand the cellular changes of the *CcNST1* overexpression lines. Evidently, the overexpression lines displayed markedly enhanced lignified vascular bundles (Fig. 5e & f), and the vascular tissue possessed more lignified cell layers and enlarged areas than did the wild type (Fig. 5c–f & i). Furthermore, the mature zone of root tissues contained many more lignified cells in the transgenic lines than the wild type (Fig. 5g–h & j). Subcellular analysis of the CcNST1-GFP fusion protein revealed that CcNST1 was localized in the nucleus (Fig. 5b). To investigate the downstream events, we analyzed the expression levels of *AtBLH6*, *AtMYB46*, and *AtMYB83* (downstream targets of *AtNST1*). All three tested genes were significantly up-regulated in the transgenic lines, while the expression of *AtNST1* went unchanged (Fig. 5k). These results indicated *CcNST1* was a potential key regulator for initiating the lignin biosynthesis pathway and that it therefore might play important roles in fruit lignification.

### Overexpression of *CcNST1* in hybrid poplar activated lignin biosynthesis

**Figure 6:**
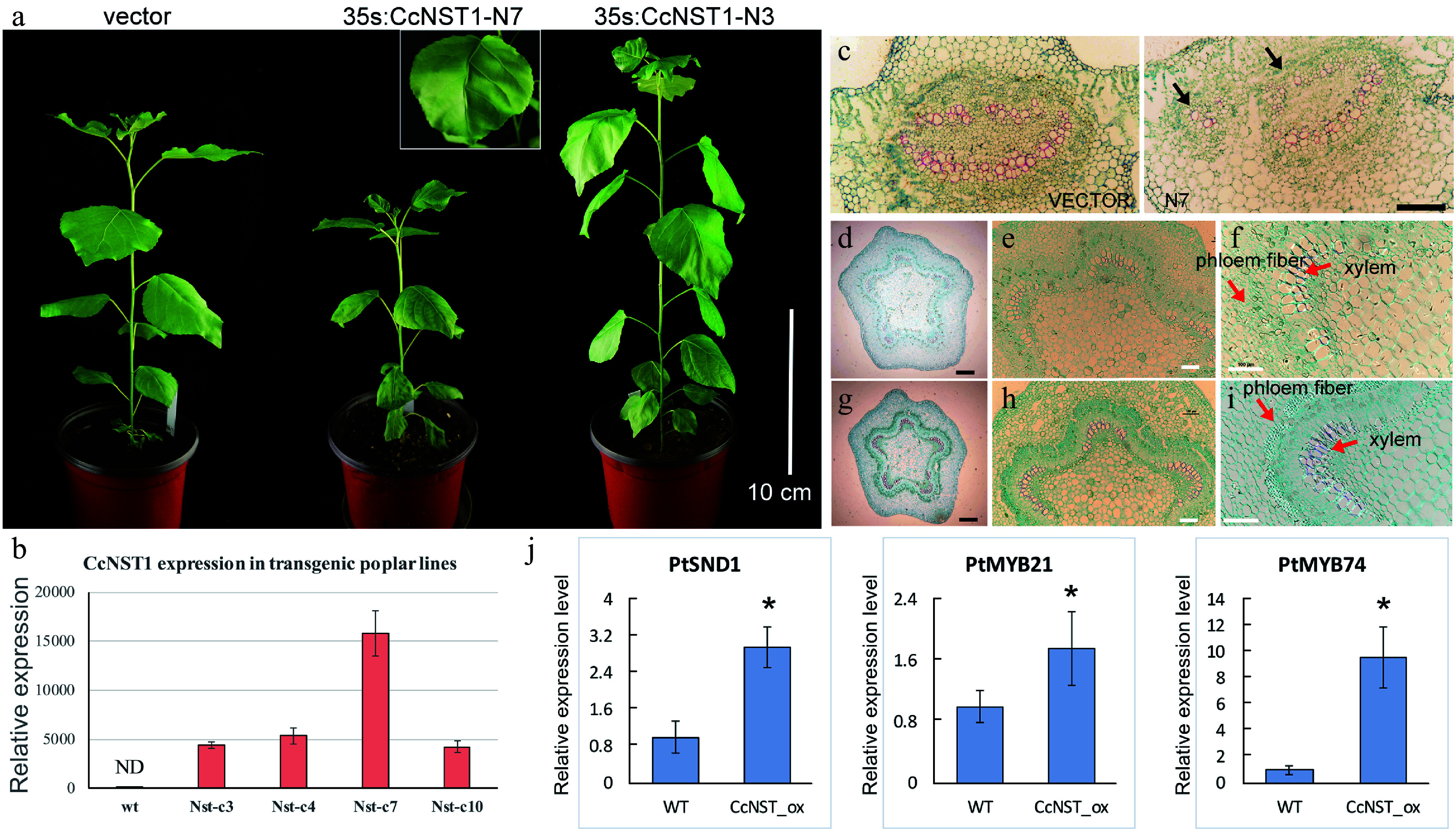
Ectopic expression of *CchNST1* in hybrid poplar (‘Nanlin 895’). (a) Comparison of overall morphology between the control and transgenic poplar plants. On the left is a transgenic plant of the empty vector as a control; middle and right are plants of independent lines of 35s:CcNST1. The inset shows a close-up view of the distorted leaf midvein in the transgenic lines. Scale bar = 10 cm. (b) Expression level of *CcNST1* in different independent transgenic lines. ND, not detected. (c) Vertical sections of the midrib in control (left) and 35s:CcNST1 (right) lines. (d)-(f) Cross sections of the fourth internode of control lines. Red arrows indicate the phloem fiber cells and xylem cells. Black scale bars = 250 µm, white scale bars = 100 µm. (g)-(i) Cross sections of the fourth internode of the 35s:CcNST1 lines. The enhanced secondary cell wall in phloem fiber cells and xylem cells are shown. Black scale bars = 250 µm, white bars 100 = µm. (j) Expression of downstream genes *PtSND1*, *PtMYB21*, and *PtMYB74* in the control and transgenic *CcNST1* lines. Three independent transgenic lines were used for gene expression analysis. Asterisks indicate a significant Student’s t-test (p < 0.05). In (b) and (j) values are means ± s.d. of three biological replicates.

The NST-type transcription factor has been shown to possess conserved functions in the model tree species *P. trichocarpa*^[16]^. Accordingly, it is interesting to know whether the function of CcNST1 is conserved across woody species. We performed a transgenic analysis using hybrid poplar ('Nanlin895') and generated overexpression lines of *CcNST1*. Expression of *CcNST1* in independent poplar lines was confirmed and the enhanced expression of *CcNST1* was detectable at the early stage of leaf development (Fig. 6b). During vegetative growth, the transgenic poplar featured consistent phenotypes, including drooping leaves and disordered leaf veins (Fig. 6a). By contrast, no obvious stem phenotypes were distinguishable. Then a histological analysis was performed to characterize the leaf midrib and stem tissue of the wild type versus overexpression lines in poplar. This showed that, transgenic line, their midribs at the distorted position had abnormal vascular tissues: some lignified cells formed irregular vascular-like tissues (Fig. 6c & d). The anatomy of stem structures was investigated further by using the fourth internode where the vascular system is established^[38]^. We found that the transgenic lines displayed enhanced lignified cells in their xylems (Fig. 6d–i), similar to the results for *A. thaliana* (Fig. 5c–f). Further, the transgenic lines evidently contained condensed parenchyma cells (Fig. 6g–i), suggesting a role for secondary cell wall formation. To evaluate the potential functioning of *CcNST1*, the downstream TFs of the *NST* homolog in *P. trichocarpa* were tested: the expression levels of *SND1*, *MYB21* and *MYB74* were all significantly up-regulated in the overexpression lines (Fig. 6j). Taken together, from these results we concluded that *CcNST1* harbors conserved functions of lignin biosynthesis and secondary cell wall formation in woody plant species.

## DISCUSSION

Tissue-specific transcriptomics analysis is widely used to identify regulators involved in plant development, growth, and responses to environmental stress^[39]^. Lignification of specific fruit tissues is an evolutionary significant process that affects seed dispersal, whose regulation is also of great economic importance in fruit crops. Although a vast number of transcriptomic studies of various fruit types have been reported in recent years, the comprehensive analysis of fruit tissue-specific transcriptomics remains relatively scarce. In the present work, we performed a detailed tissue-specific transcriptome analysis of *C. chekiangoleosa* based on its fruit lignification pattern (Fig. 1a). The DEGs’ analysis focused on those genes associated with the highly lignified EN and SC tissue, which revealed thousands of them that were potentially involved in the fruit lignification process (Fig. 1). Functional analysis suggested the enriched DEGs were related to various biological pathways including the phenylpropanoid biosynthesis pathway (Fig. 2c & d), which implicates a central role for lignin biosynthesis during fruit tissue patterning. In peach fruit, for example, a genome-wide characterization of its transcriptome during the phase of stone cell formation in endocarp found evidence for the induction of prominent phenylpropanoid, lignin, and flavonoid pathway genes^[40,41]^. Likewise, a transcriptomics study of three developmental stages of pear fruit demonstrated that up-regulation of* Cinnamoyl-CoA Reductase* (*CCR*) was involved in stone cell formation^[42]^. Our results from the gene expression analyses are largely consistent with previous work (Fig. 3), which suggests that common regulatory pathways are involved in establishing fruit lignification patterns.

The formation of a specific lignification pattern in fruits is regulated by the coordination of several types of TFs active during the developmental stages of fruit. *Camellia* plants form typical capsular fruits that undergo two independent lignification events, that of fruit peels and that of the seed coat^[31]^. A genetic model for how lignification of *C. chekiangoleosa* fruits is directed has been proposed: a cascade of TFs, starting with the SHP-FUL MADS-box TFs through to bHLH-type TFs, NAC, MYB, and BLH TFs work together to regulate the biosynthesis of the cell wall and secondary metabolites during fruit development^[40]^. We showed that the expression patterns of different types of TFs, including NAC, MYB, and BLH-like families, are correlated with the lignin accumulation in *C. chekiangoleosa* fruits (Fig. 3b). Hence, our results provided empirical evidence of the transcriptional network underlying that fruit’s lignification pattern. We also found that *Camellia* fruits are diverse in their size, secondary metabolites, and seed oil contents^[31]^. But little is known about genetic regulation of fruit development in *Camellia* species, probably because of insufficient molecular biology tools. In the future, the functional characterizations of those TFs in *C. chekiangoleosa* will be essential for elucidating the regulatory mechanism responsible for that plant’s specific lignification pattern.

Lignification is a unique process contributing critically toward the maintenance and regulation of plants growth and development and their responses to biotic/abiotic stresses. Although lignin biosynthesis and its transcriptional regulation have been extensively studied for wood formation, lignification's regulation during fruit development is not yet well characterized, especially in fruit crops. The genus *Camellia* contains many species whose seed oil production is economically valuable. The fruit lignification is also a critical breeding trait associated with fruit size, seed dispersal, and oil yield^[31]^. Recent work on* C. japonica* characterized the homolog gene of *SHP1/2* (*CjPLE*) and revealed its potential role in regulating the pattern of fruit lignification; however, based on the callus-transformation assay, direct activation of lignin biosynthesis genes by *CjPLE* was not proved^[24]^. Here we evaluated the key lignin biosynthesis genes and TFs in* C. chekiangoleosa*, finding that major lignin-related genes were highly expressed in both EN and SC tissues (Fig. 3). Therefore, we proposed that the activation of lignin biosynthesis in specified tissues requires a hierarchical interaction of TFs during fruit development.

The NST-like TFs are recognized as master regulators in the regulation of lignin biosynthesis for secondary cell wall formation in two well studied plants, *A. thaliana* and *P. trichocarpa*^[15]^. Research on fruit crops has uncovered conserved functions of homologs of NST-like NAC genes for regulating the fruit lignification process^[43]^. In loquat fruits, four NAC TFs (*EjNAC1*-*4*) are correlated with lignin accumulation in response to low temperature storage and heat stress^[27]^. Functional analyses showed that *EjNAC1* and *EjNAC3* are capable of directly activating the expression of lignin biosynthesis genes^[27]^. We found that *CcNST1* was highly expressed in EN and SC tissues, whose levels correlated with the lignification pattern (Figs 1 & 3). Further, we showed that ectopic expression of *CcNST1* in *A. thaliana* and hybrid poplar augmented tissue lignification (Figs 5 & 6). These results provided evidence that *CcNST1* acts as a positive regulator of lignin biosynthesis in *C. chekiangoleosa*. Also, in the transgenic lines of poplar, the expression of *SND1*, *MYB21*, and *MYB74 *— downstream TFs of the poplar NST gene — was significantly up-regulated (Fig. 6). This result suggests *CcNST1* is a high hierarchical activator of lignin biosynthesis during fruit development. Future work using the *Camellia*-based genetic transformation systems is now required to uncover the downstream genes regulated by *CcNST1*.

## SUPPLEMENTARY DATA

Supplementary data to this article can be found online.
