# Supplementary material for: Tissue-specific transcriptomics reveals a central role of CcNST1 in regulating the fruit lignification pattern in Camellia chekiangoleosa, a woody oil-crop
Source: For Res (Fayettev). 2022 Aug 3;2:10. doi: 10.48130/FR-2022-0010 (PMC11524261; doi:10.48130/FR-2022-0010)

Supple. Fig.2 Morphological characterizations of the growth of *Camellia chekiangoleosa* fruits. **A**, The morphology of fruit at different timepoints of fruit growth. The lignin patterns at each corresponding stage are displayed below. Bar 2 cm. **B**, A growth curve of the *C. chekiangoleosa* fruits is presented by measuring of fruit weight, transverse diameter and vertical diameter. Transverse diameter1 and transverse diameter 2 are two independent measurements with angle around 90 degrees at each sampling point of fruits.

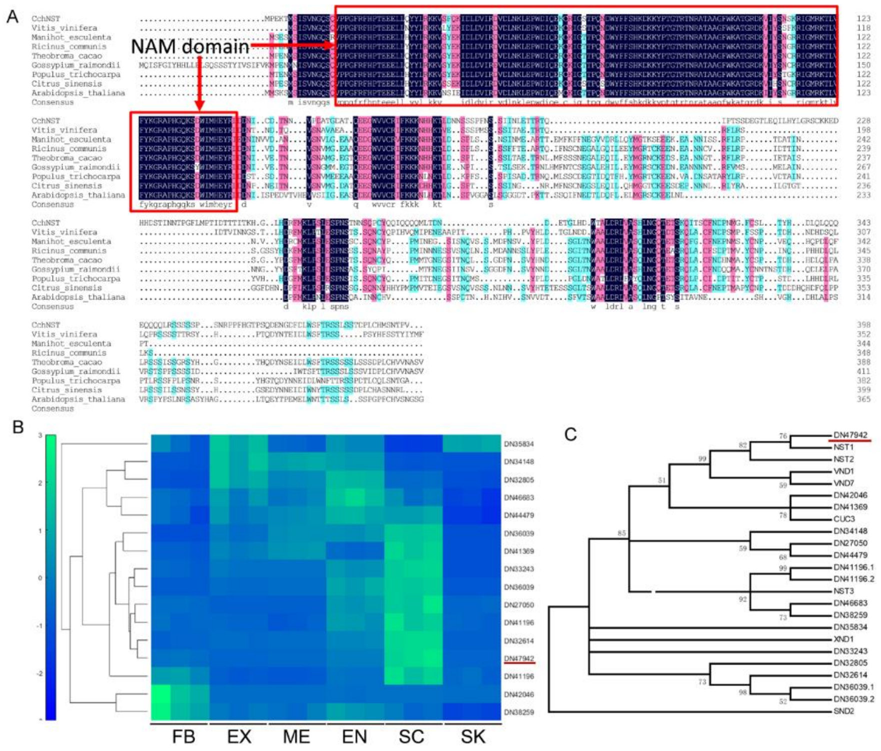

Supplement: Supplementary file 1 — Supplementary data to this article can be found online. [file FR-2022-0010-S1.zip › 10.48130_FR-2022-0010-Suppl-Figure2.pdf]
