# Supplementary material for: Tissue-specific transcriptomics reveals a central role of CcNST1 in regulating the fruit lignification pattern in Camellia chekiangoleosa, a woody oil-crop
Source: For Res (Fayettev). 2022 Aug 3;2:10. doi: 10.48130/FR-2022-0010 (PMC11524261; doi:10.48130/FR-2022-0010)

Supple. Fig.3 The identification of NST homolog in *Camellia chekiangoleosa*. **A**, The alignment of protein sequences of CcNST1 and its homologs from diverse plant species. The red rectangle indicates the conserved NAM domain. The accession numbers of sequences: *Vitis vinifera*: VV15G07370; *Manihot esculenta*: ME03581G00220; *Ricinus communis*: RC27964G00230; *Theobroma cacao*:TC0001G33230; *Gossypium raimondii*: GR01G15080; *Populus trichocarpa*: PT14G10480; *Citrus sinensis*: CS00001G04980; *Arabidopsis thaliana*: AT2G46770. **B**, The expression profiles of NST-like transcripts that are identified from the *C. chekiangoleosa* transcriptome as described in Supple. Table 2. The candidate NST ortholog was underlined by red. **C**, The phylogenic tree of NST-like genes from *Arabidopsis thaliana* and *C. chekiangoleaosa*.

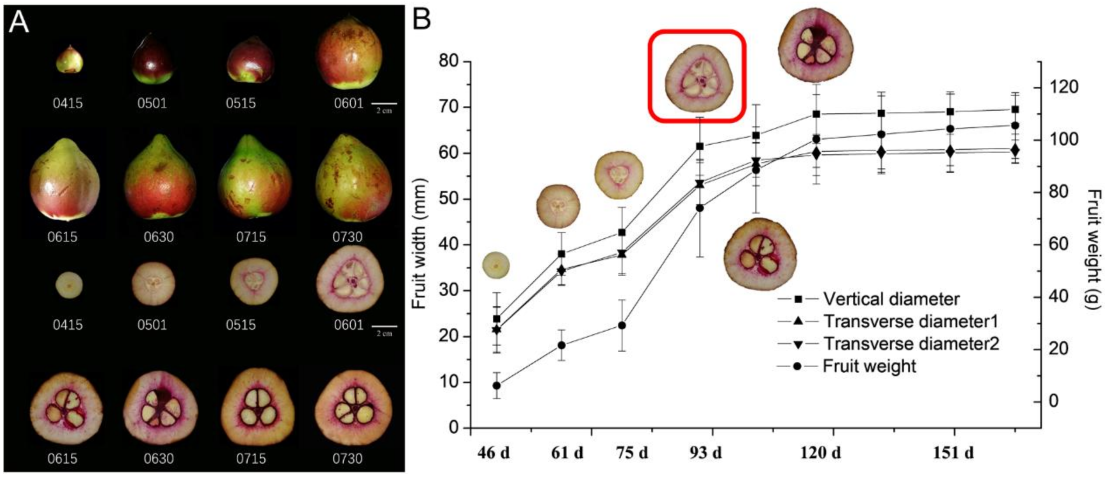

Supplement: Supplementary file 1 — Supplementary data to this article can be found online. [file FR-2022-0010-S1.zip › 10.48130_FR-2022-0010-Suppl-Figure3.pdf]
