# Supplementary material for: Tissue-specific transcriptomics reveals a central role of CcNST1 in regulating the fruit lignification pattern in Camellia chekiangoleosa, a woody oil-crop
Source: For Res (Fayettev). 2022 Aug 3;2:10. doi: 10.48130/FR-2022-0010 (PMC11524261; doi:10.48130/FR-2022-0010)

Supple. Fig.1 The evaluation of the annotation of Camellia chekiangoleosa transcriptome. **A**, The distribution of unigenes that are annotated in the Gene Ontology (GO) database. **B**, The distribution of unigenes that are annotated in the eggNOG database. C, The distribution of unigenes that are annotated in the KEGG database.

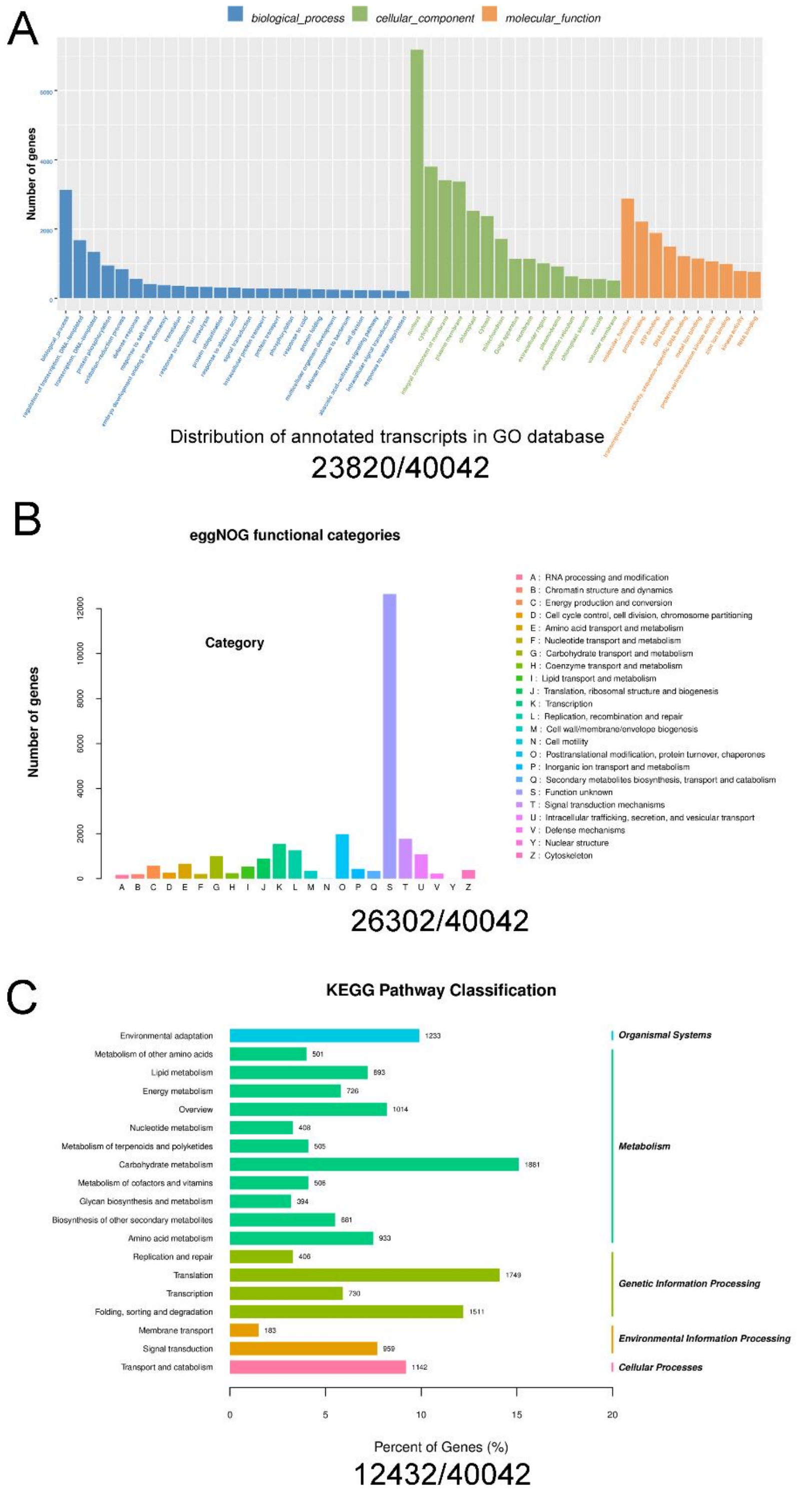

Supplement: Supplementary file 1 — Supplementary data to this article can be found online. [file FR-2022-0010-S1.zip › 10.48130_FR-2022-0010-Suppl-FigureS1.pdf]
