# Supplementary material for: Tissue-specific transcriptomics reveals a central role of CcNST1 in regulating the fruit lignification pattern in Camellia chekiangoleosa, a woody oil-crop
Source: For Res (Fayettev). 2022 Aug 3;2:10. doi: 10.48130/FR-2022-0010 (PMC11524261; doi:10.48130/FR-2022-0010)

Supple. Fig.4 The verification of transgenic Arabidopsis lines. **A**, The amplification of construct specific fragments of Arabidopsis genomic DNA. Wt, wild type; N4, N7, N10 are representative 35s:CcNST1 lines. **B**, The expression of CcNST1 in Arabidopsis lines. The wild type is not detectable (nd). Different letters (a, b) indicate significant difference by the Student's test $p < 0.05$.

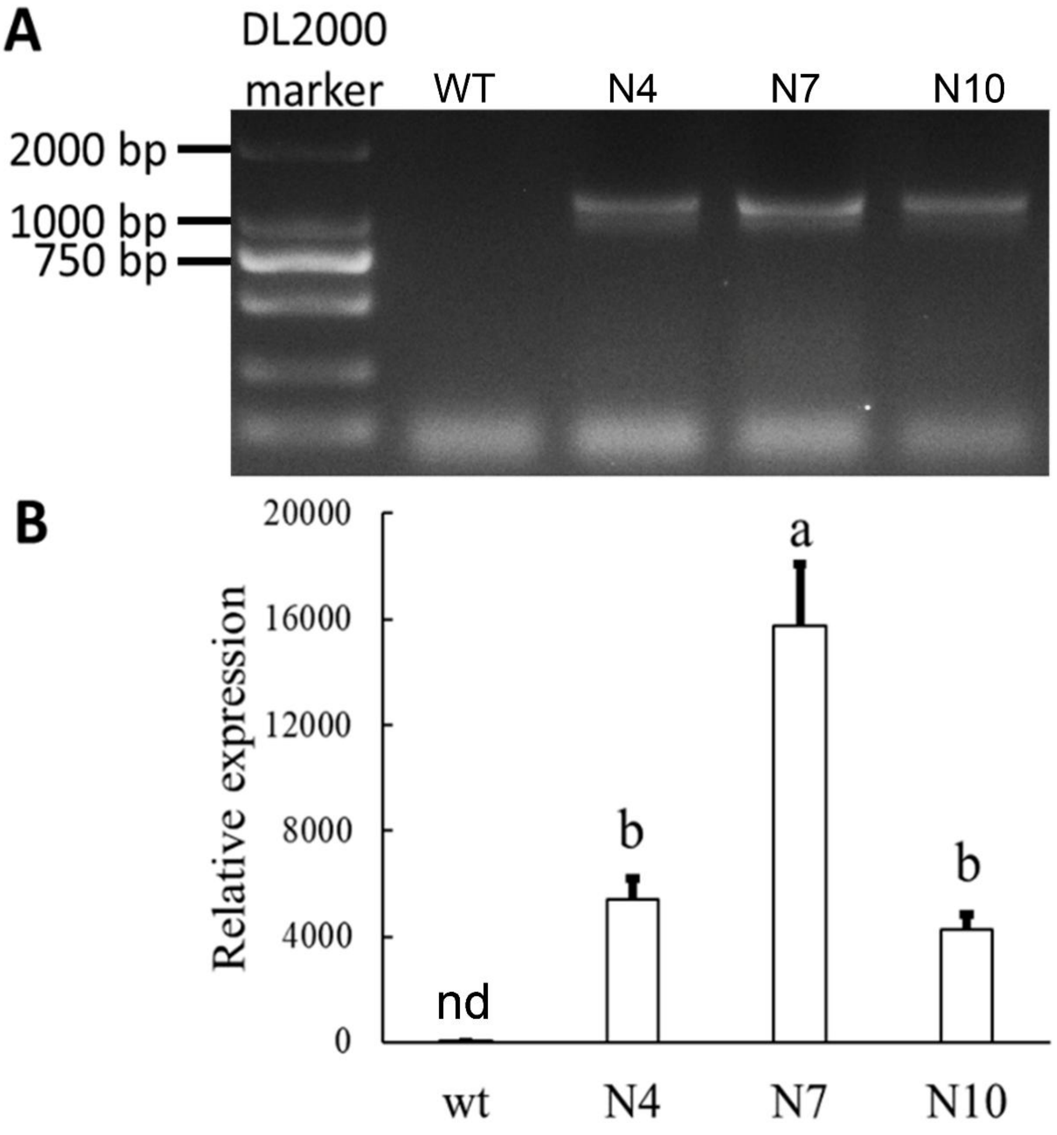

Supplement: Supplementary file 1 — Supplementary data to this article can be found online. [file FR-2022-0010-S1.zip › 10.48130_FR-2022-0010-Suppl-FigureS4.pdf]
