# Supplementary material for: Tissue-specific transcriptomics reveals a central role of CcNST1 in regulating the fruit lignification pattern in Camellia chekiangoleosa, a woody oil-crop
Source: For Res (Fayettev). 2022 Aug 3;2:10. doi: 10.48130/FR-2022-0010 (PMC11524261; doi:10.48130/FR-2022-0010)
Supplement: Supplementary file 1 — Supplementary data to this article can be found online. [file FR-2022-0010-S1.zip › 10.48130_FR-2022-0010-Suppl-TableS1.pdf]

Supplementary Information: this file includes 5 supplementary tables and 4 supplementary figures with legends.

Supplementary Table 1. Primers used in this study.

Primer	Usage	Forward Sequences (5-3)	Reverse Sequences (5-3)
**CchNST1-clone**	Full-length Amplification	TGTGTAAGCTAGCTCTATCCGA	ACGTATCGTCCAATATTGAAAAACTCT
**CchNST1-EX**	Gateway cloning	tcagcagtcgaagagcATGCCAGAAAAAACCATGAG	ttagcgtgtgaagagcTACAGGAGTGTTGGACATA
**CchNST1-Q**	qRT-PCR	GCAGGATGAAAATGGCGATT	TCGGTAGAAGACAAGGAAGATCGT
**CchTUB-Q**	Reference for Camellia chekiangoleosa Qpcr	CCGGGCGGTGATCTTG	TGATCAATGCGCGAGAACA
**AtActin-qF**	Reference for Arabidopsis thaliana Qpcr	AGAGATTCAGATGCCCAGAAGTCTTGTTCC	AACGATTCCTGGACCTGCCTCATCATACTC
**CchNST1-Pd**	construct-specific of 35sCcNST:GFP	GGATCCatttggagagaacacggggg	CTGCAGacatgcttaacgtaattcaacaga
**PdSND1-Q**	Qpcr in Populus	TCAGCTTAATGGCCAGACTGAA	ATAAGTAATGGTTGGGTCAATGCA
**PdMYB21-Q**	Qpcr in Populus	GCATTTTCACCCCAAGAAGA	CCGCAATTTGAGACCACCTA
**PdMYB74-Q**	Qpcr in Populus	TTTTGGGTAACAGGTGGGCT	CTTCTCCTCTTTCACCCCCAC
**PdActin-Q**	Reference for Populus trichocarpa Qpcr	AGAGATTCAGATGCCCAGAAGTCTTGTTCC	AACGATTCCTGGACCTGCCTCATCATACTC
**cch-PAL-Q**	TRINITY_DN33936_c0_g2	CGCCTCGCGGGTATCA	CAAGGCCCTCTTTAGGCTGTAA
**cch-C4H-Q**	TRINITY_DN37968_c1_g1	CGTGGTGGCTTGCAAACA	CTCGGGCCTGAACTCTTCTG
**cch-4CL-Q**	TRINITY_DN39894_c1_g1	TCAGGCGGACGAGAAGAATG	CACGACATCGTCTGGGTTGA
**cch-C3H-Q**	TRINITY_DN28133_c0_g3	CACATTCCGTGGCTTCGTT	TGCACCATGCTTGGCAAA
**cch-HCT-Q**	TRINITY_DN46437_c2_g1	CCACCCCAGCCTGCTTT	GCGGCTTTCATGGAAGGA
**cch-COMT-Q**	TRINITY_DN24253_c2_g4	ACACCAAGCTTGCGACTAAGAAT	CGGGTTGTGAGCCAACATG
**Cch-CCoAOMT-Q**	TRINITY_DN38542_c0_g1	ATGCCCCGCTCAGGAAGTAT	AAGGCCTTGTTCAGCTCCAA
**cch-F5H3-Q**	TRINITY_DN40625_c1_g3	GATGTTTGGCGGCATTGAA	CCGCCATTGACCATTCGA
**cch-CCR-Q**	TRINITY_DN27918_c1_g2	AACCCAGTATTGGTGCTTGGA	CATGAATAATGCTCGCGTTGA
**cch-CAD-Q**	TRINITY_DN41222_c2_g1	TCCAATTTCTCACCCCTGTTG	CCGATGAAGCTCCCTGTTATG
**cch-LACC1-Q**	TRINITY_DN29772_c1_	TGTTCACTACTGACTTTCCGGGTAA	GGTGGAGTGCCCGTGTAATT

	g3		
**cch-POX-Q**	TRINITY_DN24750_c2_g5	CCAGCTCCCACCTTCAACAT	TGGACAGACCTCTTTGGGAGAA
**cch-BLH6-Q**	TRINITY_DN40632_c0_g1	GATTACCTCGTCTCCGCTATGTG	AAACTGTTGAAGCGCCTTTTTT
**cch-MYB46-Q**	TRINITY_DN36951_c0_g4	CCTCTCGACAACGCCTACGA	TCCCAACCATGTCGGTATGTT
**cch-MYB85-Q1**	TRINITY_DN35568_c1_g3	AATCACAGAAAACTGACAATGGAATC	CAGAGGCGAGCACGATGA
**cch-MYB85-Q3**	TRINITY_DN33357_c4_g2	TGGTCCAAGATTGCTTCTCATCT	TGGGTATTCCAATGGTTCTTGAT
**cch-MYB85-Q4**	TRINITY_DN38430_c0_g7	CGTTCCGTTCCAATCCCTAA	TCTCCGCCGAGTTGGAGTT
**cch-MYB85-Q5**	TRINITY_DN24116_c0_g6	CCGCCACATGGCACAAT	TCCTTTGAAAGTCGTGCTTCAG
**cch-MYB103-Q2**	TRINITY_DN24116_c0_g1	CCATCACCGCCACGAAAT	TGGTATTGTCAATGGCATGGA
**cch-MYB58**	TRINITY_DN40512_c1_g1	TCCTATGGCAACAATGAAGATACAG	CTTGTTGGCCAGACCTTCTTG
