# Supplementary material for: Tissue-specific transcriptomics reveals a central role of CcNST1 in regulating the fruit lignification pattern in Camellia chekiangoleosa, a woody oil-crop
Source: For Res (Fayettev). 2022 Aug 3;2:10. doi: 10.48130/FR-2022-0010 (PMC11524261; doi:10.48130/FR-2022-0010)
Supplement: Supplementary file 1 — Supplementary data to this article can be found online. [file FR-2022-0010-S1.zip › 10.48130_FR-2022-0010-Suppl-TableS2.pdf]

Supplementary Table 2. Identification of NAC domain transcription factors of *Camellia chekiangoleosa* transcriptome based on BLAST search. In total, 15 transcripts were found to contain complete open reading frame (ORF).

TranscriptID	ORF type	ORF Length(aa)	CDS region	BLASTP eValue
TRINITY_DN47942_c0_g19	complete	399	93-1289(+)	2.00E-70
TRINITY_DN29065_c0_g6	3prime_partial	197	323-910(+)	5.00E-70
TRINITY_DN40903_c1_g3	3prime_partial	176	360-884(+)	8.00E-67
TRINITY_DN42046_c1_g3	complete	352	97-1152(+)	3.00E-45
TRINITY_DN41369_c1_g4	complete	173	473-991(+)	1.00E-43
TRINITY_DN27050_c2_g1	complete	341	562-1584(+)	3.00E-41
TRINITY_DN29065_c0_g7	5prime_partial	557	88-1080(+)	5.00E-40
TRINITY_DN44479_c0_g1	complete	331	283-1845(+)	1.00E-39
TRINITY_DN32805_c0_g2	complete	521	1-600(+)	2.00E-39
TRINITY_DN31682_c0_g2	5prime_partial	200	2-1672(+)	3.00E-38
TRINITY_DN36039_c1_g8	complete	169	128-634(+)	4.00E-38
TRINITY_DN36039_c1_g6	complete	349	128-1174(+)	6.00E-38
TRINITY_DN41196_c0_g5	complete	175	90-614(+)	8.00E-38
TRINITY_DN41196_c0_g3	complete	158	403-876(+)	8.00E-38
TRINITY_DN46683_c0_g1	complete	525	305-1879(+)	2.00E-37
TRINITY_DN32614_c1_g4	complete	336	137-1144(+)	4.00E-37
TRINITY_DN40858_c1_g4	5prime_partial	305	1-915(+)	3.00E-36
TRINITY_DN34148_c0_g1	5prime_partial	210	2-631(+)	2.00E-35
TRINITY_DN32642_c5_g1	3prime_partial	127	26-403(+)	5.00E-34
TRINITY_DN34148_c0_g3	complete	281	65-907(+)	7.00E-34
TRINITY_DN27962_c3_g5	3prime_partial	148	101-541(+)	1.00E-33
TRINITY_DN38259_c0_g1	complete	628	173-2056(+)	7.00E-31
TRINITY_DN27200_c1_g1	5prime_partial	265	3-797(+)	1.00E-29
TRINITY_DN33738_c0_g1	5prime_partial	365	1-1095(+)	6.00E-28
TRINITY_DN26004_c2_g7	5prime_partial	211	2-634(+)	1.00E-24
TRINITY_DN35834_c1_g2	complete	174	364-885(+)	4.00E-24
TRINITY_DN33243_c2_g2	complete	207	762-1382(+)	3.00E-22
TRINITY_DN33243_c2_g4	3prime_partial	105	577-888(+)	1.00E-21
TRINITY_DN49765_c2_g1	3prime_partial	151	491-940(+)	1.00E-20
