# Supplementary material for: Tissue-specific transcriptomics reveals a central role of CcNST1 in regulating the fruit lignification pattern in Camellia chekiangoleosa, a woody oil-crop
Source: For Res (Fayettev). 2022 Aug 3;2:10. doi: 10.48130/FR-2022-0010 (PMC11524261; doi:10.48130/FR-2022-0010)
Supplement: Supplementary file 1 — Supplementary data to this article can be found online. [file FR-2022-0010-S1.zip › 10.48130_FR-2022-0010-Suppl-TableS3.pdf]

Supplementary Table 3. **An overview of the RNA-seq statistics of *Camellia chekiangoleosa* tissues**. Each tissue type includes three biological replicates. Q20 and Q30 represent base call accuracy of 99% and 99.9% respectively. FB, Floral Bud; EX, Exocarp; ME, Mesocarp; EN, Endocarp; SC, Seed Coat; SK, Seed Kernel.

Sample	Raw Reads	Raw Bases	Clean Reads	Clean Bases	Valid rate (%)	Q20 (%)	Q30 (%)	GC (%)
**FB_1**	66697194	10.07G	64344974	9.47G	96.47	98.23	95.03	45.27
**FB_2**	84246372	12.72G	81454180	11.97G	96.69	98.10	94.81	45.39
**FB_3**	86757252	13.10G	83820638	12.34G	96.62	98.24	95.02	45.57
**EN_1**	79572064	12.02G	77454800	11.40G	97.34	98.16	94.89	45.54
**EN_2**	87383270	13.19G	85400092	12.58G	97.73	98.24	95.02	46.14
**EN_3**	81941748	12.37G	80176182	11.80G	97.85	98.21	94.97	46.04
**SK_1**	81256296	12.27G	79404974	11.68G	97.72	98.17	94.88	45.53
**SK_2**	75136956	11.35G	72810172	10.71G	96.90	98.20	94.96	45.76
**SK_3**	85564586	12.92G	83440540	12.28G	97.52	98.21	94.97	45.98
**EP_1**	80838750	12.21G	77915068	11.50G	96.38	98.40	95.29	46.07
**EP_2**	74659820	11.27G	72095568	10.66G	96.57	98.45	95.36	47.24
**EP_3**	85281996	12.88G	82237258	12.15G	96.43	98.44	95.35	46.54
**ME_1**	78968354	11.92G	76791370	11.35G	97.24	98.42	95.30	47.12
**ME_2**	68368758	10.32G	66657086	9.85G	97.50	98.44	95.35	46.96
**ME_3**	81224454	12.26G	79565026	11.76G	97.96	98.44	95.38	45.93
**SC_1**	81520820	12.31G	79128248	11.69G	97.07	98.41	95.31	45.28
**SC_2**	81302620	12.28G	78885922	11.64G	97.03	98.32	95.12	45.91
**SC_3**	66648220	10.06G	65223778	9.64G	97.86	98.45	95.44	45.51
**Avg.**	79298307							
