# Supplementary material for: Tissue-specific transcriptomics reveals a central role of CcNST1 in regulating the fruit lignification pattern in Camellia chekiangoleosa, a woody oil-crop
Source: For Res (Fayettev). 2022 Aug 3;2:10. doi: 10.48130/FR-2022-0010 (PMC11524261; doi:10.48130/FR-2022-0010)
Supplement: Supplementary file 1 — Supplementary data to this article can be found online. [file FR-2022-0010-S1.zip › 10.48130_FR-2022-0010-Suppl-TableS4.pdf]

Supplementary Table 4. **The statistics of *Camellia chekiangoleosa* transcriptome**. The transcripts and unigenes are obtained by using the Trinity v2.11.0 software. Sequences less than 200 bp are removed.

	All	GC%	Min Length（bp）	Median Length（bp）	Max Length（bp）	Total Assembled Bases（bp）	N50（bp）
**Transcript**	206367	40.79	201	684	13728	202547343	1492
**Unigene**	40042	40.85	201	835	13728	44816160	1676
