# Supplementary material for: Tissue-specific transcriptomics reveals a central role of CcNST1 in regulating the fruit lignification pattern in Camellia chekiangoleosa, a woody oil-crop
Source: For Res (Fayettev). 2022 Aug 3;2:10. doi: 10.48130/FR-2022-0010 (PMC11524261; doi:10.48130/FR-2022-0010)
Supplement: Supplementary file 1 — Supplementary data to this article can be found online. [file FR-2022-0010-S1.zip › 10.48130_FR-2022-0010-Suppl-TableS5.pdf]

Supplementary Table 5. The identification of genes related to lignin biosynthesis and transcriptional regulation based on the information from Arabidopsis thaliana (TAIR v.11).

GENE	AGI CODE	HOMOLOGOUS GENE CODE	SCORE	E-VALUE	DEG
**LIGNIN BIOSYNTHESIS GENE**					
**PAL1**	AT2G37040	TRINITY_DN48964_c1_g1	143	7.00E-33	YES
		TRINITY_DN33936_c0_g2	117	4.00E-25	NO
		TRINITY_DN48964_c0_g4	119	1.00E-25	YES
		TRINITY_DN33936_c0_g3	117	4.00E-25	YES
		TRINITY_DN48964_c0_g1	68	3.00E-10	YES
**C4H**	AT2G30490	TRINITY_DN37968_c1_g1	133	5.00E-30	NO
		TRINITY_DN36925_c1_g3	105	1.00E-21	YES
**4CL1**	AT1G51680	TRINITY_DN34501_c0_g6	62	2.00E-08	YES
		TRINITY_DN39894_c1_g1	52	2.00E-05	NO
**C3H1**	AT2G40890	TRINITY_DN28133_c0_g3	76	1.00E-12	YES
**HCT**	AT5G48930	TRINITY_DN46437_c2_g1	107	2.00E-22	YES
**CSE**	AT1G52760	TRINITY_DN34444_c0_g4	52	9.00E-06	NO
**COMT**	AT5G54160	TRINITY_DN24253_c2_g4	178	7.00E-44	YES
**CCOAOMT1**	AT4G34050	TRINITY_DN25789_c0_g2	256	2.00E-67	YES
		TRINITY_DN38542_c0_g1	170	1.00E-41	YES
**F5H1**	AT4G36220	TRINITY_DN40625_c1_g3	98	3.00E-19	NO
**CCR1**	AT1G15950	TRINITY_DN27918_c1_g2	78	2.00E-13	NO
**CAD3**	AT4G34230	TRINITY_DN41222_c2_g1	161	2.00E-38	NO
**LAC4**	At2g38080	TRINITY_DN29772_c1_g3	133	5.00E-30	YES
		TRINITY_DN45270_c1_g4	54	4.00E-06	NO
		TRINITY_DN44491_c1_g2	54	4.00E-06	YES
		TRINITY_DN36780_c0_g2	54	4.00E-06	YES
**PRX2**	AT1G05250	TRINITY_DN24750_c2_g5	50	4.00E-05	YES
**LIGNIN TRANSCRIPTIONAL REGULATION GENE**					
**NST1**	AT2G46770	TRINITY_DN47942_c0_g19	139	6.00E-32	YES
**MYB46/83**	AT5G12870/	TRINITY_DN36951_c0_g4	105	6.00E-22	YES
**MYB85/42**	AT4G22680/	TRINITY_DN35568_c1_g3	178	5.00E-44	NO
	AT4G12350	TRINITY_DN26557_c1_g12	143	3.00E-33	YES
		TRINITY_DN24116_c0_g6	72	8.00E-12	NO
		TRINITY_DN38430_c0_g7	56	5.00E-07	NO
**MYB58/63**	AT1G16490/	TRINITY_DN40512_c1_g1	107	2.00E-22	NO
**MYB103**	AT1G63910	TRINITY_DN24116_c0_g1	78	2.00E-13	NO
**BLH**	AT4G34610	TRINITY_DN40632_c0_g1	121	2.00E-26	NO
